# Hybrid reference-based Video Source Identification

**DOI:** 10.3390/s19030649

**Published:** 2019-02-05

**Authors:** Massimo Iuliani, Marco Fontani, Dasara Shullani, Alessandro Piva

**Affiliations:** 1 Department of Information Engineering, University of Florence, 50139 Florence, Italy; massimo.iuliani@gmail.com (M.I.); shullani.dasara@gmail.com (D.S.); 2 FORLAB—Multimedia Forensics Laboratory, PIN Scrl, 59100 Prato, Italy; 3 Amped Software, Loc. Padriciano 99, 34149 Trieste, Italy; marco.fontani@ampedsoftware.com

**Keywords:** image forensics, video forensics, social media, sensor pattern noise, smartphone, video database

## Abstract

Millions of users share images and videos generated by mobile devices with different profiles on social media platforms. When publishing illegal content, they prefer to use anonymous profiles. Multimedia Forensics allows us to determine whether videos or images have been captured with the same device, and thus, possibly, by the same person. Currently, the most promising technology to achieve this task exploits unique traces left by the camera sensor into the visual content. However, image and video source identification are still treated separately from one another. This approach is limited and anachronistic, if we consider that most of the visual media are today acquired using smartphones that capture both images and videos. In this paper we overcome this limitation by exploring a new approach that synergistically exploits images and videos to study the device from which they both come. Indeed, we prove it is possible to identify the source of a digital video by exploiting a reference sensor pattern noise generated from still images taken by the same device. The proposed method provides performance comparable with or even better than the state-of-the-art, where a reference pattern is estimated from video frames. Finally, we show that this strategy is effective even in the case of in-camera digitally stabilized videos, where a non-stabilized reference is not available, thus solving the limitations of the current state-of-the-art. We also show how this approach allows us to link social media profiles containing images and videos captured by the same sensor.

## 1. Introduction

Digital videos (DVs) are steadily becoming the preferred means for people to share information in an immediate and convincing way. Recent statistics show a 75% increase in the number of DVs posted on Facebook in one year [1] and posts containing DVs yield more engagement than their text-only counterparts [2]. Interestingly, most of this content is captured using smartphones, whose impact on digital photography is dramatic: in 2016 compact camera sales consistently dropped worldwide, mostly because they are being replaced by smartphones, which are always at your fingertips and make sharing much easier [3].

In such a scenario, it is not surprising that DVs gained importance also from the forensic and intelligence point of view: videos have recently been used to spread terror over the web, and many critical events have been filmed and shared by thousands of web users. In such cases, investigating the digital history of DVs is of paramount importance in order to recover relevant information, such as acquisition time and place, authenticity, or information about the source device. Recently, Multimedia Forensics tools have been developed for such tasks, based on the observation that each processing step leaves a distinctive trace on the digital content, as a sort of digital fingerprint. By detecting the presence, the absence or the incongruence of such traces it is possible to blindly investigate the digital history of the content [4].

In particular, the source identification problem—that is, univocally linking the digital content to the device that captured it—has received great attention in the last years. Currently, the most promising technology to achieve this task exploits the detection of the Sensor Pattern Noise (SPN) left by the acquisition device [5]. This footprint is universal (every sensor introduces one) and unique (two SPNs are uncorrelated even in case of sensors belonging to cameras of the same brand and model). As far as still images are concerned, SPN has been proven to be robust to common processing operations like JPEG compression [5], or even uploading to Social Media Platforms (SMPs) [6,7].

In contrast, research on source device identification for DVs is not as advanced. This is probably due to the higher computational and storage requirements for video analysis, the use of different video coding standards, and the absence of sizeable datasets available to the community for testing. Indeed, DV source identification borrowed both the mathematical background and the methodology used for still images [8,9]: in a similar way, assessing the origin of a DV requires the analyst to have either the source device or some training DVs captured by that device, from which to extract the reference SPN.

However, if we consider that 85% of shared media are captured using smartphones, which use the same sensor to capture both images and videos, it is possible to exploit images also for video source identification. A first attempt at using still images to estimate the video fingerprint was recently described in [10], where authors noticed how image and video patterns of some portable devices, acquiring non-stabilized video, can be generally related by cropping and scaling operations. However, in the research community, there is still no better way to perform image and video source identification than computing two different reference SPNs, one for still images and one for videos. In addition, a strong limitation is represented by the presence in many mobile devices of in-camera DV stabilization algorithms. In this case, a non-stabilized SPN reference cannot be estimated from a DV since each frame possibly exploits a different physical portion of the camera sensor.

The first contribution of this work is a hybrid source identification approach that exploits still images for estimating a fingerprint that can be used to verify the source of a video. To this aim, the geometrical relation between image and video acquisition processes are studied for 18 modern smartphones, including devices featuring in-camera digital stabilization. As a second contribution we prove that the proposed technique, while preserving the state of the art performance for non-stabilized videos, is able to effectively detect the source of in-camera digitally stabilized videos as well. Furthermore, we show that this hybrid approach allows linking images and videos downloaded from different social media platforms, namely Facebook and YouTube.

The rest of the paper is organized as follows: Section 2 introduces SPN based source device identification, and reviews the state of the art for DV source identification; Section 3 formalizes the considered problem and describes the proposed hybrid approach; Section 4 presents the video dataset used in the tests, and discusses some YouTube/Facebook technical details related to the SPN; Section 5 focuses on the experimental validation of the proposed technique—including results obtained on stabilized videos and content exchanged through SMPs—and a comparison with existing approaches; finally, Section 6 draws some final remarks and outlines future works.

Everywhere in this paper, vectors and matrices are indicated in bold as X and their components as X(i) and X(i,j) respectively. All operations are element-wise, unless mentioned otherwise. Given two vectors X and Y, ||X|| is the euclidean norm of X, X·Y is the dot product between X and Y, $\bar{X}$ is the mean value of X, $\rho (s_{1},s_{2};X,Y)$ is the normalized cross-correlation between X and Y calculated at the spatial shift $\left(s_{1},s_{2}\right)$ as

$$\begin{array}{c} \rho \left(s_{1},s_{2};X,Y\right)=\frac{\sum_{i}\sum_{j}\left(X\left[i,j\right]-\bar{X}\right)\left(Y\left[i+s_{1},j+s_{2}\right]-\bar{Y}\right)}{||X-\bar{X}||||Y-\bar{Y}\left|\right|}, \end{array}$$

where the shifts [i,j] and $\left[i+s_{1},j+s_{2}\right]$ are taken modulo the horizontal and vertical image dimensions. If X and Y dimensions mismatch, a zero down-right padding is applied. Furthermore, we denote its maximum by $\rho_{peak}\left(X,Y\right)=\rho \left(s_{peak};X,Y\right)=\max_{s_{1},s_{2}}\rho \left(s_{1},s_{2};X,Y\right)$. The notations are simplified in $\rho (s_{1},s_{2})$ and in $\rho_{peak}$ when the two vectors cannot be misinterpreted.

## 2. Digital Video Source Device Identification Based on Sensor Pattern Noise

The task of blind source device identification has gathered great attention in the multimedia forensics community. Several approaches were proposed to characterize the capturing device by analysing traces like sensor dust [11], defective pixels [12], and color filter array interpolation [13]. A significant breakthrough was achieved when Lukas et al. [5] first introduced the idea of using Photo-Response Non-Uniformity (PRNU) noise to univocally characterize the camera sensor. Being a multiplicative noise, PRNU cannot be effectively removed even by high-end devices; moreover, it remains in the image even after JPEG compression at average quality. The suitability of PRNU-based camera forensics for images retrieved from common SMPs has been investigated in [6], showing that modifications applied either by the user or by the SMP can make the source identification based on PRNU ineffective. The problem of scalability of SPN-based camera identification has been investigated in several works [14,15]. Noticeably, in [14] authors showed that the Peak-to-Correlation Energy (PCE) provides a significantly more robust feature compared to normalized correlation. The vast interest in this research field fostered the creation of reference image datasets specifically tailored for the evaluation of source identification [16], allowing a thorough comparison of different methods [17]. Recently, the authors of [18] addressed the problem of reducing the computational complexity of fingerprint matching, both in terms of time and memory, through the use of random projections to compress the fingerprints, at the price of a small reduction in matching accuracy. Sensor noise is also exploited in [19] to address the problem of clustering a set of images based on their source device. All the methods mentioned so far have been conceived for (and tested on) still images. Although research on image and video source identification began almost at the same time, the state of the art of the latter is much poorer. In their pioneering work [8], Chen et al. proposed extracting the SPN from each frame separately and then merging the information through a Maximum Likelihood Estimator; the PCE was recommended [8] for the fingerprint matching phase. Experimental results show that resolution and compression have an impact on performance, but identification is still possible if the number of considered frames can be increased (10 minutes for low resolution, strongly compressed videos). Two years later, Van Houten et al. investigated the feasibility of camcorder identification with videos downloaded from YouTube [20], yielding encouraging results: even after YouTube recompression, source identification was possible. However, results in [20] are outdated, since both acquisition devices and video coding algorithms have evolved significantly since then. This study was extended by Scheelen et al. [21], considering more recent cameras and codecs. Results confirmed that source identification is possible; however, the authors clarify that the reference pattern was extracted from reference and natural videos before re-encoding. Concerning reference pattern estimation, Chuang et al. [22] firstly proposed treating the SPN extracted from video frames differently based on their encoding type; the suggested strategy is to weigh differently intra- and inter-coded frames, based on the observation that intra-coded frames are less compressed and thus more reliable for PRNU fingerprint estimation. A recent contribution from Chen et al. [23] considered video surveillance systems, where the videos transmitted over an unreliable wireless channel can be affected by blocking artifacts, complicating pattern estimation.

Most of the research on video forensics neglects the analysis of digitally stabilized videos, where the SPN can hardly be registered. In [24] an algorithm was proposed to compensate the stabilization on interlaced videos. However, the method was tested on a single device and it is inapplicable on the vast majority of modern devices, that are equipped with a 1080p camera (where *p* stands for *progressive*). Recently, Taspinar et al. [10] showed that digital stabilization applied out of camera by a third party program can be managed by registering all video frames on the first frame through rotation and scaling transformation. However, the technique is proved to be effective only when a reference generated from non-stabilized videos is available. This is a gap to be filled considering that most modern smartphones feature in-camera digital stabilization, and that in many cases such a feature cannot be disabled.

It should be noted that all the mentioned works discuss source identification either for still images or videos (with the only exception of [10]), and in the vast majority of cases the reference pattern is estimated from “clean” contents, i.e., images or frames as produced by the device, without any alteration due to re-encoding or (even worse) upload/download from SMPs. This approach seriously limits the applicability of source device identification, since it assumes that a specific kind of media (videos or still images) are available to the analyst. In the following sections we show how to exploit the available mathematical frameworks to determine the source of a DV based on a reference derived by still images, even in the case of in-camera digitally stabilized videos, and eventually how to apply this strategy to link images and videos from different SMPs.

## 3. Hybrid Sensor Pattern Noise Analysis

**Figure 1:**
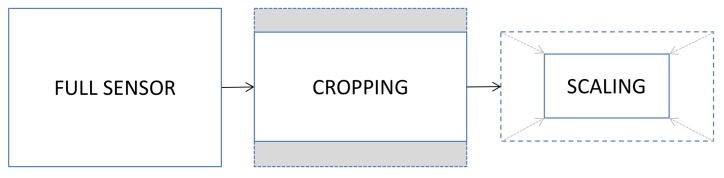
Workflow of the geometric transformation applied in a video acquisition.

DVs are commonly captured at a much lower resolution than images: top-level portable devices reach 4K video resolution at most (which means, 8 Megapixels per frame), while the same devices easily capture 20 Megapixels images. During video recording, a central crop is carried out to adapt the sensor size to the desired aspect ratio (commonly 16:9 for videos), then the resulting pixels are scaled to match exactly the desired resolution (see Figure 1). As a direct consequence, the sensor pattern noise extracted from images and the one extracted from videos cannot be directly compared and, most of the time, it is not sufficient to just scale them to the same resolution because of cropping.

The Hybrid reference-based Source Identification (HSI) process consists of identifying the source of a DV based on a reference derived from still images. The strategy involves two main steps: (i) the reference fingerprint is derived from still images acquired by the source device; (ii) the query fingerprint is estimated from the investigated video and then compared with the reference to verify the possible match.

Moreover, the camera fingerprint K can be estimated from *N* still images (or frames) $I^{\left(1\right)},\ldots ,I^{\left(N\right)}$ captured by the source device. A denoising filter [5,25] is applied to each frame and the noise residuals $W^{\left(1\right)},\ldots ,W^{\left(N\right)}$ are obtained as the difference between each frame and its denoised version. Then the fingerprint estimation $\tilde{K}$ is derived by the maximum likelihood estimator [26]:

(1) $$\tilde{K}=\frac{\sum_{i=1}^{N}W^{\left(i\right)}I^{\left(i\right)}}{\sum_{i=1}^{N}{\left(I^{\left(i\right)}\right)}^{2}}.$$

The fingerprint of the video query is estimated in the same way from the available video frames. Let $K_{I}$ and $K_{V}$ be the image and video fingerprints, $m_{I}\times n_{I}$ and $m_{V}\times n_{V}$ their resolution, ${\tilde{K}}_{I}=K_{I}+\Xi_{I}$ and ${\tilde{K}}_{V}=K_{V}+\Xi_{V}$ their correspondent esteems from still images and video frames where $\Xi_{I}$ and $\Xi_{V}$ are noise terms. According to Section 2 and to Figure 1, if the two fingerprints belong to the same camera sensor, we expect the existence of a 2D isometric transformation that maps the elements of $K_{I}$ to those of $K_{V}$, i.e., a scale 0<s≤1 and a translation $\left(t_{x},t_{y}\right)\in N^{2}$ exist such that:

(2) $$\begin{array}{c} K_{I}\left(si+t_{x},sj+t_{y}\right)=K_{V}\left(i,j\right),\\ \forall i=1,\ldots m_{I},\;\mathrm{with}\;m_{I}>m_{V},\\ \forall j=1,\ldots n_{I},\;\mathrm{with}\;n_{I}>n_{V}. \end{array}$$

Note that we are assuming that the video fingerprint is at a smaller resolution than the image fingerprint since it represents the default camera settings in most mobile devices nowadays. The model still holds in the opposite scenario. If we denote such a transformed fingerprint with $K_{I}^{\sigma }$, the source identification can be formulated as a two-channel hypothesis testing problem [27]:

(3) $$\begin{array}{c} H_{0}:K_{I}^{\sigma }\neq K_{V},\;\forall \sigma ,\\ H_{1}:K_{I}^{\sigma }=K_{V},\;\exists \sigma . \end{array}$$

Then, the test statistic is built as proposed in [28], where the problem of camera identification from images that were simultaneously cropped and resized was studied: the two-dimensional normalized cross-correlation $\rho (s_{1},s_{2})$ is calculated for each of the possible spatial shift $\left(s_{1},s_{2}\right)$ determined within a set of feasible cropping parameters. Then, given the peak $\rho_{peak}$, its sharpness is measured by the PCE ratio [14] as:

(4) $$PCE=\frac{\rho {\left(s_{peak}\right)}^{2}}{\frac{1}{mn-|V|}\sum_{s\notin V}\rho {\left(s\right)}^{2}}$$

where V is a small set of peak neighbours, $m=\max(m_{I},m_{V})$ and $n=\max(n_{I},n_{V})$.

In order to consider the possible different scaling factors of the two fingerprints—since videos are usually resized—a brute force search can be conducted considering the PCE as a function of *L* plausible scaling factors $r_{1},\ldots r_{L}$. Then its maximum

(5) $$P=\max_{r_{i},\;i\in \left[1,L\right]}PCE\left(r_{i}\right),$$

is used to determine whether the two fingerprints belong to the same device. Practically, if this maximum overcomes a threshold τ, $H_{1}$ is decided and the corresponding values $s_{peak}$ and $r_{peak}$ are exploited to determine the cropping and the scaling factors. In [28] it is shown that a theoretical upper bound for False Alarm Rate (FAR) can be obtained as

(6) $$FAR=1-{\left(1-Q\left(\sqrt{\tau }\right)\right)}^{k},$$

where Q(x)=Pr(N(0,1)>x), being N(0,1) a normal distribution, and *k* is the number of tested scaling and cropping parameters. This method is expected to be computationally expensive, especially for large dimension images. This problem can be mitigated considering that:

- the resize and cropping factors are determined by the device model (and, possibly, firmware). It is thus possible to build a lookup table that eliminates the need for an exhaustive search when information about the reference model is available;
- even when no information about the model is available, it is not necessary to repeat the whole search on all frames. Once a sufficiently high correlation is found for a given scale, the search can be restricted around it.

Section 4 shows the cropping and scaling factors, computed accordingly to this method, for 18 devices.

### 3.1. Source Identification of Digitally Stabilized Videos

Recent devices feature digital stabilization as a means to reduce the impact of shaky hands on captured videos. By estimating the impact of user movements, the software adaptively determines which portion of the sensor should be used to obtain a stable image. Image stabilization can usually be turned on and off by the user on devices based on Android, while in iOS devices this option cannot be modified in the native camera application. Source identification of videos captured with active digital stabilization cannot be accomplished using the classical approach of PRNU fingerprint computation, since it would require the fingerprint to remain spatially aligned across all frames. This condition is not met due to the stabilization process [29]. HSI solves the problem on the reference side (the fingerprint is estimated from still images) but the issue remains on the query side. A first way to compensate digital stabilization was proposed in [24] and tested on a single Sony device. Recently, in [10], it was proposed to compute the fingerprint from a stabilized video by using the first frame noise pattern as reference, and registering all following frames on such reference by estimating the similarity transformation that maximizes the correlation between the patterns. The technique was proved to compensate for digital stabilization applied off camera by third party software with limited reliability, probably because the reference for the whole process is computed from a single frame. In the HSI paradigm, however, still images are exploited to estimate a more reliable fingerprint, while on the query side each video frame is registered on the image reference based on a similarity transformation. In Section 5.3 we will show that, using this technique, reliable source identification is possible even when using only the first five frames of digital stabilized videos obtained from modern devices. In the next section, we define a hybrid source identification pipeline conceived to reduce false alarm probability and computational effort.

### 3.2. Hybrid Reference-Based Video Source Identification Pipeline

**Figure 2:**
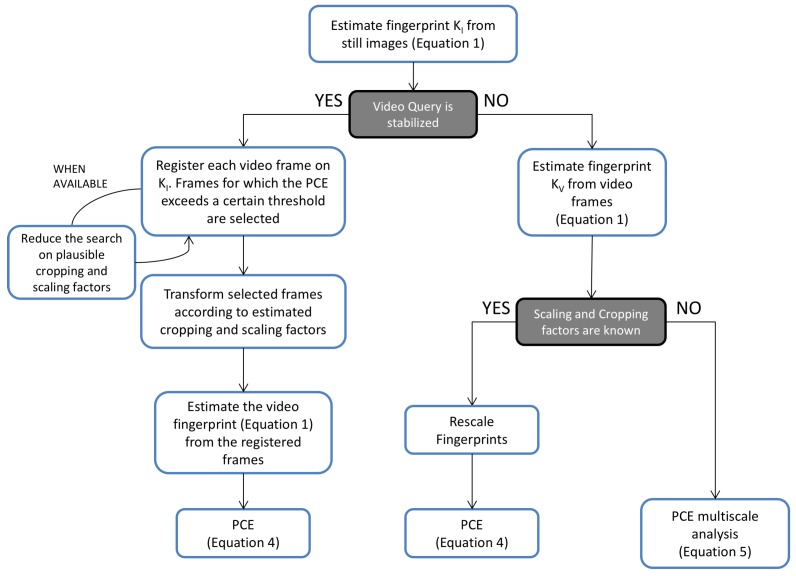
Hybrid reference-based Source Identification (HSI) pipeline to source attribution of a query video.

Given a query video and a set of images belonging to a reference device, the proposed pipeline is summarized in Figure 2.

First, the device fingerprint $K_{I}$ is estimated from still images according to Equation (1). Then, stabilized videos are preliminarily identified by splitting the frames in two groups that are used independently to estimate two different fingerprints, as described in [10], and by computing their PCE; a low PCE value will expose the presence of digital stabilization. If no stabilization is detected, the video fingerprint $K_{V}$ is estimated by treating video frames as still images. Conversely, each frame is registered on the reference $K_{I}$ searching for plausible parameters based on PCE values. In case the expected range of parameters is known, the search can be reduced to save computational effort and mitigate the false alarm probability (see Section 5.1 for details). Only registered video frames for which the PCE exceeds a threshold τ are then aggregated to estimate the video fingerprint $K_{V}$. Once both fingerprints $K_{I}$ and $K_{V}$ are available, their PCE is computed and the correlation value is compared to a threshold in order to decide between $H_{0}$ and $H_{1}$, as defined in Equation (3).

### 3.3. Extension to Contents Shared on Social Media Platforms

The proposed technique can be applied to match multimedia contents exchanged through different SMPs. Let us consider a user, “Bob”, that publishes videos with criminal content to a SMP using an anonymous profile. At the same time, Bob is leading his virtual social life on another social network, where he publicly shares his everyday pictures. Unaware of the traces left by the sensor, he captures with the same device the contents shared on both profiles. In such a scenario, the fingerprints derived from the images and videos on the two social platforms may be compared with the proposed method to link Bob to the criminal videos.

Analysing multimedia contents shared on SMPs is not a trivial task. Indeed, besides stripping all metadata, SMPs usually re-encode images and videos. For example, Facebook policy is to down-scale and re-compress images so to obtain a target bit-per-pixel value [30]; YouTube also scales and re-encodes DVs [31]. Needless to say, forensic traces left in the signal are severely hindered by such processing. Fortunately, the sensor pattern noise is one of the most robust signal-level features, surviving down-scaling followed by compression. Nevertheless, when it comes to link the SPN extracted from, say, a YouTube video and a Facebook image, a new problem arises: since both content have been scaled/cropped by an unknown factor, such transformations must be estimated in order to align the patterns.

**Figure 3:**
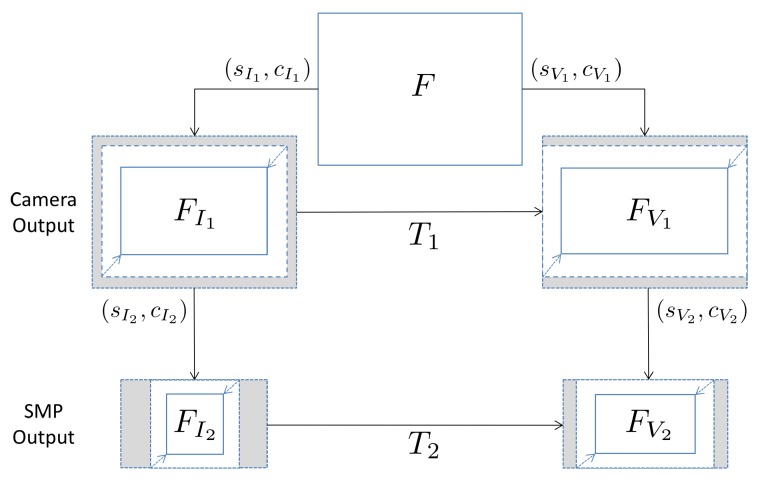
Workflow of the geometric transformation applied to the sensor noise. From the full frame *F*, an image $F_{I1}$ or video $F_{V1}$ is acquired by the camera, and exchanged through a Social Media Platform (SMP) creating $F_{I2}$ or $F_{V2}$.

Interestingly, the hybrid approach can be applied to this scenario. In Figure 3 the geometric transformations occurring on the contents are summarized, starting from the full frame *F*: during acquisition, the image $F_{I_{1}}$ is produced scaling and cropping *F* by factors $s_{I_{1}}$ and $c_{I_{1}}$, respectively. Uploading to the SMP causes a new transformation—with factors $s_{I_{2}}$ and $c_{I_{2}}$—leading to image $F_{I_{2}}$. Similarly, the video $F_{V_{1}}$ is generated from the camera and $F_{V_{2}}$ is uploaded to another SMP—with cropping and scaling factors of $s_{V_{1}}$, $c_{V_{1}}$ and $s_{V_{2}}$, $c_{V_{2}}$ respectively. It can be easily deduced that, for both native and uploaded contents, image and video fingerprints are linked by a geometric transformation consisting of a combination of cropping and scaling. Then, the hybrid approach that we used to determine the transformation $T_{1}$, which aligns the fingerprints of two native contents, can be also applied to determine $T_{2}$, thus directly linking $F_{I_{2}}$ to $F_{V_{2}}$. Two main drawbacks are expected for this second application. Firstly, the compared contents have probably been compressed twice, causing a degradation of the signal. Furthermore, since metadata are commonly stripped by SMPs, it may be hard to build a lookup table for scaling and cropping parameters: in this scenario, an exhaustive search across all plausible scaling and cropping factors is needed. In Section 5.4 the proposed application is tested to link videos of a YouTube profile to images of a Facebook profile.

### 3.4. Extension to Digital Zoom

Modern devices allow the user to adjust the zooming factor before or even during video acquisition. This is commonly implemented by using a sub-portion of the device sensor, possibly followed by some form of scaling to match the desired output resolution. Therefore, if a video is captured using digital zoom, an additional geometric transformation step must be considered in the HSI process. Following the same reasoning of Section 3.3, we argue that, even in the case of acquisition with digital zoom, an exhaustive search over all plausible scaling and cropping factors can successfully match the query video to the reference fingerprint obtained from images. However, different strategies should be implemented depending on whether the zoom is set to a fixed value for the whole length of the video or it varies during acquisition: in the former case, multiple frames can be combined to estimate the sensor pattern noise (provided digital stabilization is not enabled), while in the latter case each frame must be matched separately. Noticeably, since the geometrical transformations involved in these steps are linear, even zooming followed by upload and download from a social media platform would not break the detection scheme, provided the video is not re-encoded aggressively. We will experimentally verify these intuitions on a sample case in Section 5.5.

## 4. Dataset for HSI

We tested the proposed technique on a subpart of the VISION dataset [32], consisting of 1978 flat field images, 3311 images of natural scenes and 339 videos captured by 18 devices from different brands (Apple, Samsung, Huawei, Microsoft, Sony). The dataset also provides the corresponding Facebook images, in both low quality (LQ) and high quality (HQ), and the corresponding YouTube videos. These contents have been used for testing the performance on the social media platforms. The tests have been carried out on a subpart of the VISION Dataset [32] since the computational time needed to test all the devices was too high. We considered both smartphones and tablets depicting pictures and videos acquired with the default device settings that, for some models, include the automatic DV stabilization (see the VISION Dataset [32] for details). Table 1 summarizes the considered models, their standard image and video resolution and whether the digital stabilization was active on the device. From now on we will refer to these devices with the names C1,…, C18 as defined in Table 1. For each device we considered at least:

- On the reference side: 100 flat-field images depicting skies or walls; 150 images of indoor and outdoor scenes; 1 video of the sky captured with slow camera movement, longer than 10 s;
- On the query side: videos of flat surfaces, indoor scenes and outdoor scenes. For each of the video categories (flat, indoor and outdoor) at least 3 different videos have been captured considering the three different scenarios available in the Dataset: (i) still camera, (ii) walking operator and (iii) panning and rotating camera. We will refer to them as *still*, *move* and *panrot* videos respectively. Thus, each device has at least 9 videos, each one lasting more than 60 s.

## 5. Experimental Validation

The experimental section consists of five parts, each focused on a different contribution of the proposed technique:

1. We determine the cropping and scaling parameters applied by each device model in the considered set;
2. We verify that, in the case of non-stabilized video, the performance of the hybrid approach is comparable with the source identification based on a video reference;
3. We show the effectiveness in identifying the source of in-camera digitally stabilized videos;
4. We show the performance in linking Facebook and YouTube profiles;
5. We demonstrate the effectiveness of the method in the presence of digital zoom.

### 5.1. Fingerprints Matching Parameters

The scaling and cropping factors applied by each device were derived by registering the reference video fingerprint ${\tilde{K}}_{V}$ on a reference fingerprint ${\tilde{K}}_{I}$ derived from still images according to the *P* statistic (Equation (5)). For each device, we estimated ${\tilde{K}}_{I}$ by means of 100 images randomly chosen from the flat-field pictures. For non-stabilized videos, ${\tilde{K}}_{V}$ was derived by means of the first 100 frames of the reference video available for that device. We opted for using all frames of the video instead of limiting to intra-coded frames (I-frames) only; this choice may slightly limit the performance in the non-stabilized video case, but it helps to greatly reduce the computational effort in the stabilized video case. Indeed, registration parameters of two consecutive frames in a stabilized video are expected to be closer than the ones of two distant I-frames; on the contrary, using only I-frames would force us to reboot the brute force search each time. In our implementation, once registration parameters are found on a frame, they are used to initialize the parameters in the next frame exploiting their proximity in time. Table 2 reports the estimated cropping parameters (in terms of coordinates of the upper-left corner of the cropped area along *x* and *y* axes, whereas the right down corner is derived by the video size) and the scaling factor, maximizing the PCE. For instance, C1 image fingerprint should be scaled by a factor of 0.59 and cropped on the upper left side of 307 pixels along the *y* axis to match the video fingerprint; C9 is a pretty unique case in which the full frame is applied for video and is left and right cropped by 160 pixels to capture images.

In the case of stabilized videos, the cropping and scaling factors change in time, with possible rotation applied too. For these devices we thus determined the registration parameters of the first 10 frames of the available video reference; the main statistics are reported in Table 3.

The information provided in Table 3 can be exploited to reduce the parameter search space in case of source identification of digitally stabilized videos. Indeed, an exhaustive search of all possible scaling and rotation parameters, required in a blind analysis, would be intractable on a large scale: in our tests a totally blind search can take up to 10 min per frame on a modern average-powered computer, while the informed search reduces the time to less than a minute for stabilized videos and a few seconds for non-stabilized videos.

### 5.2. Hybrid Reference-Based Video Source Identification Performance

**Figure 4:**
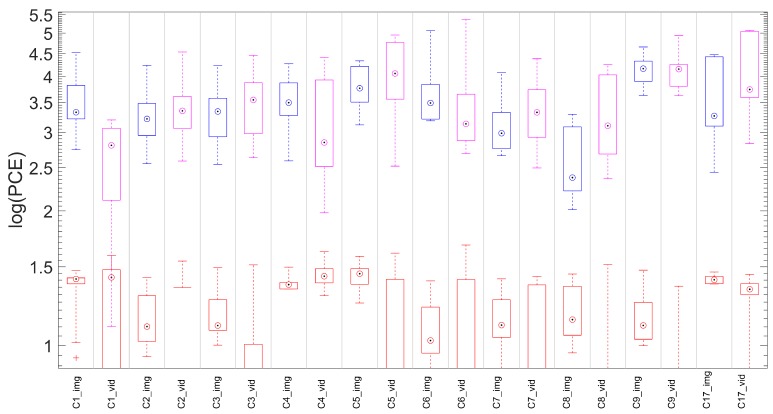
(Best viewed in color) Matching statistics $mP_{I}$ and $mP_{V}$ are represented by the blue and pink boxplots, respectively. Corresponding mismatching statistics in red. On each box, the central mark indicates the median, and the bottom and top edges of the box indicate the 25th and 75th percentiles, respectively. The whiskers denote the minimum and maximum of the statistics. For plotting purposes, we defined log(a)=−∞,∀a≤0.

In this section we compare the proposed technique with the state of the art approach [8], where the fingerprint is derived by estimating the SPN from a reference video. The comparison is only meaningful for non-stabilized devices. For each device, the reference fingerprints ${\tilde{K}}_{I}$ and ${\tilde{K}}_{V}$ were derived respectively from the first 100 natural reference images (for the proposed method) and from the first 100 frames of the reference video (for the video reference approach). Given a video query, the fingerprint to be tested was derived by the first 100 frames and compared with ${\tilde{K}}_{V}$ and with ${\tilde{K}}_{I}$ while using the cropping and scaling parameters expected for the candidate device (Equation (4)). We refer to the test statistics as $P_{V}$ and $P_{I}$ to distinguish the reference origin (video frames or still images). For each device, we tested all the available matching pairs (i.e., reference and query from the same source device) and an equal number of mismatching pairs (i.e., reference and query from different source devices) randomly chosen from all available devices. We refer to these statistics as $mP_{I}$ and $mmP_{I}$ respectively ($mP_{V}$ and $mmP_{V}$ for video references). In Figure 4 we report for each device: (i) the statistics $mP_{I}$ and $mP_{V}$ (blue and pink respectively) of matching pairs; (ii) ${\bar{mmP}}_{I}$ and ${\bar{mmP}}_{V}$ (in red), the statistics for mismatching cases.

The plot shows that distributions can be perfectly separated when the reference is estimated from images (100% accuracy), while in the video reference case the accuracy is 99.5%, confirming that performance are comparable.

### 5.3. Hybrid Reference-Based Video Source Identification Performance on Stabilized Videos

State of the art results in identifying the source of a stabilized video are provided in [10]. The authors, based on a similar registration protocol, analyze the performance using both non-stabilized and stabilized references. Their results are reported in Table 4 for convenience: we see that, if a non-stabilized reference is available, the method achieves a True Positive Rate (TPR) 0.83 at a 0 False Positive Rate (FPR). Unfortunately, in several modern devices (e.g., Apple smartphones) digital stabilization cannot be turned off without third party applications; in this case, only stabilized reference can be exploited, achieving a TPR of 0.65.

We will now show that this performance drop can be solved by exploiting the proposed HSI method.

**Figure 5:**
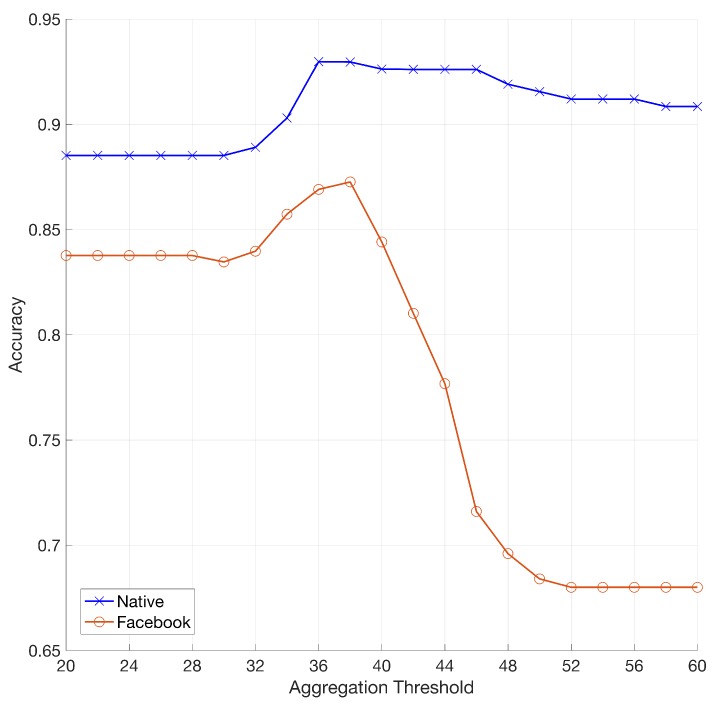
(Best viewed in color) Mean accuracy of source identification on digitally stabilized videos by varying the aggregation threshold τ. Native and Facebook (HQ) contents are referred in blue and orange respectively.

**Figure 6:**
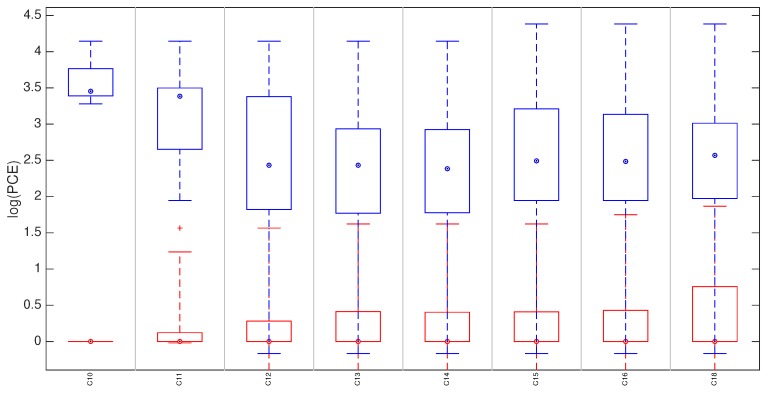
(Best viewed in color) Details of the performance achieved with best aggregation threshold (38) on native stabilized videos. Matching and mismatching statistics are reported in blue and red, respectively, for each device.

For each device, the reference fingerprints ${\tilde{K}}_{I}$ was estimated from 100 natural images. Given a video query, each frame is registered on ${\tilde{K}}_{I}$ searching within the expected parameters for the candidate device (as derived in Section 5.1). The video fingerprint ${\tilde{K}}_{V}$ is then obtained by aggregating all registered video frames whose PCE with ${\tilde{K}}_{I}$ overcomes the aggregation threshold τ. Finally, the aggregated fingerprint ${\tilde{K}}_{V}$ is compared with the reference SPN ${\tilde{K}}_{I}$. All tests were performed limiting the analysis to the first 5 frames of each video. For each device, we tested all available matching videos and an equal number of mismatching videos randomly chosen from all available devices. In Figure 5 we show the system accuracy by varying the aggregation threshold τ. Table 5 shows, for different values of τ, the TPR and FPR corresponding to the best accuracy. Figure 6 shows the matching and mismatching PCE statistics obtained using τ=38.

Results clearly show that, using τ=50, the system achieves a TPR equal to 0.83, which is totally consistent with results achieved in [10], but in our case without the need for a non-stabilized video reference. Moreover, results show that using a slightly lower aggregation threshold some improvement can be achieved, (TPR 0.86 with an aggregation threshold of 38).

It is important to point out that in our experiments the achieved results required a significant computational time, indeed finding a frame matching (overcoming the proposed threshold) took between 2.7 s and 10 h. The computer used for the experimental validation is an Intel(R) Core(TM) i7-6700 CPU @ 3.40GHz with 32 GB of RAM and running Ubuntu 18.04.1 LTS; our Matlab implementation of the multiscale analysis between image and video reference is available on the DINFO website (https://lesc.dinfo.unifi.it/it/node/184). The runtime depends on several factors, such as the reference and the query resolution, the availability of accurate matching parameters (see Section 5.1) and the video content. However, the mean time required to manage a single test on 5 frames, was around 45 min, thus less than 10 min per frame. Notice that the availability of accurate matching parameters (see Section 5.1) allowed us to perform mismatching tests with the same computational time: when the tested parameters exceed the expected range for that device, the test ends with a negative matching. A full search on all available scaling and rotations parameters is intractable.

### 5.4. Results on Contents from SMPs

**Figure 7:**
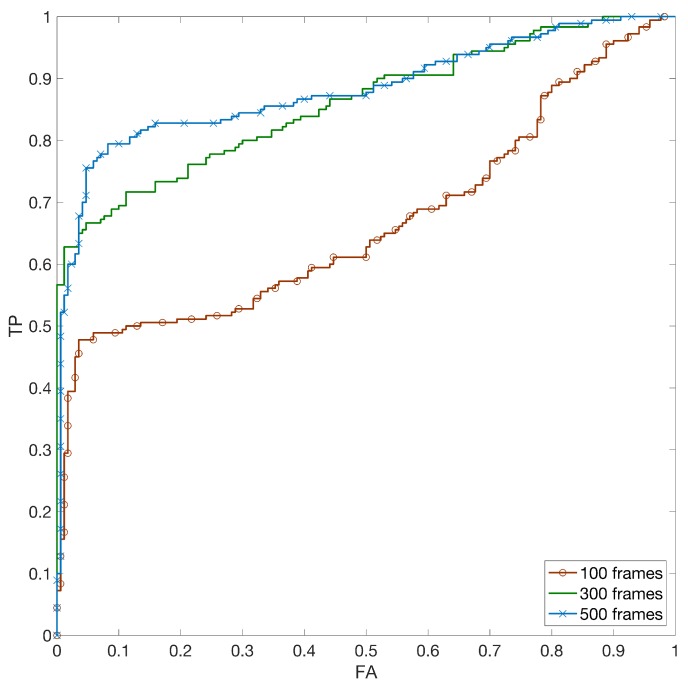
(Best viewed in color) Receiver Operating Characteristic (ROC) curve for profile linking between non-stabilized YouTube videos and Facebook high quality (HQ) images by varying the number of frames to estimate the video reference.

In this section we test the HSI approach in the application scenario of linking Facebook and YouTube accounts containing images and videos captured with the same device. For clarity, we considered the non-stabilized and stabilized cases separately. Furthermore, we conducted two experiments: one estimating the SPN from images uploaded to Facebook using the high-quality option, and another experiment estimating the SPN from images uploaded using the low quality option. A detailed explanation of the differences between the two options is given in [30], here we only mention the fact that under low-quality upload images are downscaled so that their maximum dimension does not exceed 960 pixels, while under high-quality upload the maximum allowed dimension rises to 2048 pixels. Throughout all tests, we used 100 natural images for estimating the camera fingerprint. After estimating image and video fingerprints according to the method described in the previous sections, we investigated the matching performance by varying the number of frames employed to estimate the fingerprint of the query video. For the sake of simplicity, we reported the aggregated results with a Receiver Operating Characteristic (ROC) curve, where TPR and FPR are compared, and we used the Area Under Curve (AUC) as an overall index of performance. Similarly to the previous experiment, we considered all available matching videos for each device (minimum 9 videos, 17 on average) and an equal number of randomly selected mismatching videos. In Figure 7 we report the results of the first experiment (high quality Facebook reference vs. YouTube non-stabilized videos) by using 100, 300 and 500 frames to estimate the fingerprint from the video. It can be noticed that a hundred frames is rarely enough to correctly link two profiles. Increasing the number of frames from 100 to 300 significantly improves the performance, and a minor further improvement can be achieved by increasing from 300 to 500 frames. The AUC values for the three cases are 0.67, 0.86, 0.88, respectively.

**Figure 8:**
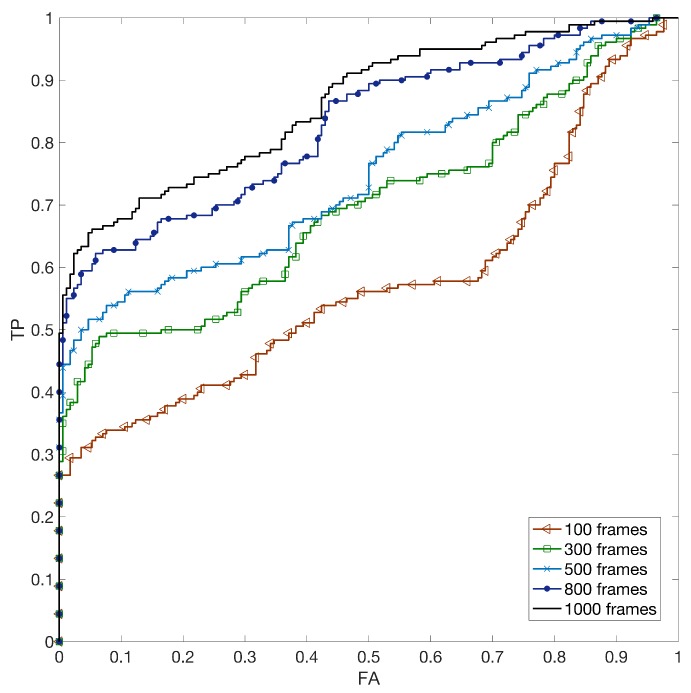
(Best viewed in color) ROC curves for profile linking between non-stabilized YouTube videos and Facebook low quality (LQ) images by varying the number of frames to estimate the video reference.

When Facebook images uploaded in low quality are used as reference, the estimated pattern is expected to be less reliable than for the high quality case. This degradation on the reference side can be mitigated by using more robust estimates on the query side; for this reason, for the low quality case, we also considered using 500, 800 and 1000 frames for extracting the query pattern, achieving ROC curves reported in Figure 8. The corresponding AUC values are 0.57,0.70,0.75,0.83 and 0.86 using 100,300,500,800 and 1000 frames respectively.

**Figure 9:**
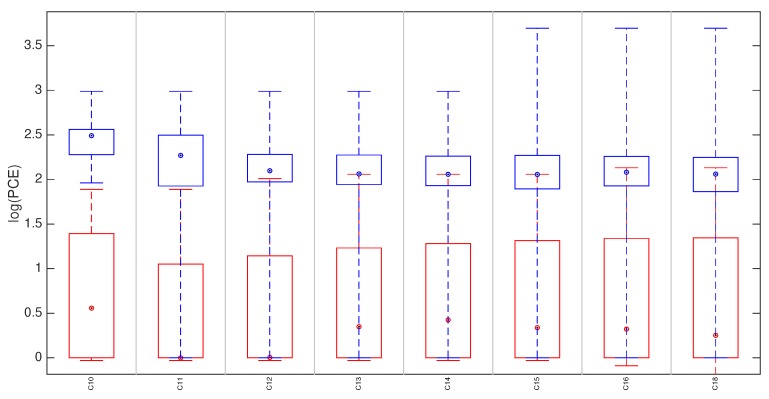
(Best viewed in color) Details of the performance achieved with best aggregation threshold (38) on stabilized YouTube videos using Facebook (HQ) images as references. Matching and mismatching statistics are reported in blue and red, respectively, for each of the devices.

We now focus on the case of in-camera stabilized videos downloaded from YouTube. Figure 5 reports the performance achieved for different values of the aggregation threshold τ. The plot suggests that using τ=38 remains the best choice also in this experiment, leading to 87.3% overall accuracy. Figure 9 details the performance for each device by applying such aggregation threshold. Thus, we can say that the hybrid approach to source identification provides promising results for linking SMP profiles even in the case of in-camera digitally stabilized videos.

### 5.5. Results on Digitally Zoomed Videos

As a final experiment, we tested the validity of the proposed approach in a sample case where a video is obtained using digital zoom. First, we concentrated on the case of a static digital zoom followed by uploading and downloading from YouTube. We selected device C17 and captured a flat video at default resolution (1080p), setting a slight amount of digital zoom before initiating the recording (the device interface does not show the actual zooming factor); the obtained video was then uploaded to YouTube and downloaded at two resolutions, namely 1080p and 720p. Then, we applied the HSI framework to match these videos to the device fingerprint obtained from 100 flat images, and used the first 150 frames of the video for query fingerprint computation. Table 6 shows that the hybrid approach was able to properly match the video even in presence of digital zoom combined with downscaling applied by YouTube during download. As a further confirmation that the measured scaling factor is reliable, we notice that 1080/720=1.5=1.3085/0.8722, which means that the detected scale closely reflects the downscaling applied by YouTube.

**Figure 10:**
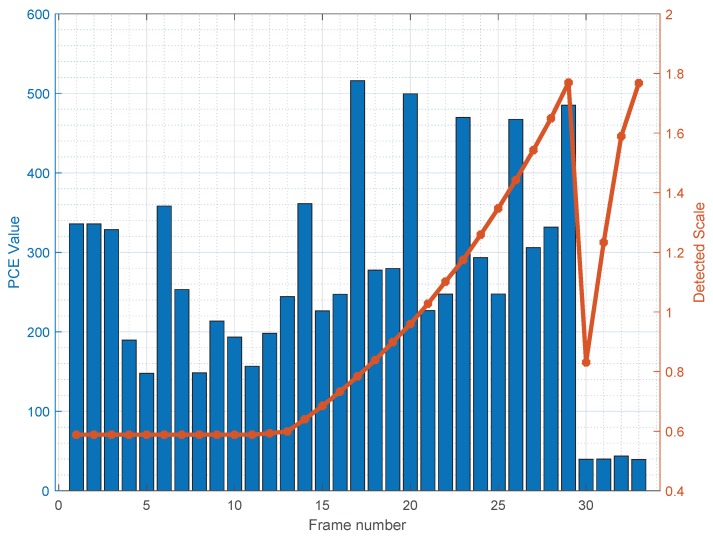
Detected scale factor, and corresponding Peak-to-Correlation Energy (PCE) value, for a video captured increasing the digital zoom from roughly half the video on.

We also tested the case of a flat video captured while adjusting the zoom factor during the acquisition (dynamic digital zoom): this situation is more challenging, because each frame of the video has to be matched separately. Even though, the hybrid approach successfully matched most of the examined frames (we limited the analysis to the first 33 frames of the video): Figure 10 shows the best PCE value and the corresponding scale as a function of the frame number. It is worth noticing that initially the detected scale remains constant (when the user has not activated the zoom) and then grows while the users zooms until the PCE drops significantly, likely because the necessary scaling exceeds the allowed range for device C17. This experiment shows that the proposed approach has promising applications to the case of zoomed videos; a thorough investigation of this aspect is left for future work.

## 6. Conclusions

In this paper we proposed an hybrid approach to video source identification using a reference fingerprint derived from still images. We showed that, in the case of non-stabilized videos, the hybrid approach yields performance comparable with or even better than the current state-of-the-art strategy, which uses a video to compute the reference pattern. As a major contribution, our approach allows reliable source identification even for videos produced by devices that enforce digital in-camera stabilization (e.g., all recent Apple devices), for which a non-stabilized reference is not available. We reported the geometrical relationships between image and video acquisition process of 18 different devices, even in case of digitally stabilized videos. The proposed method was applied to link image and video contents belonging to different social media platforms: its effectiveness has been proved to link Facebook images to YouTube videos, with promising results even in the case of digitally stabilized videos. Specifically, when low quality Facebook images are involved, we showed that some hundreds of video frames are required to effectively link the two sensor pattern noises. We finally showed that presence of digital zoom can be handled with our approach. The main limitation of the proposed approach is the need for a brute force search for determining scale (and, in the case of stabilized devices, rotation) when no information on the tested device is available. A possible way to mitigate this problem would be to design SPN descriptors that are simultaneously invariant to crop and scaling. This challenging task is left for future work.

## Figures and Tables

**Table 1:** Considered devices with their default resolution settings for image and video acquisition.

ID	Model	Image Resolution	Video Resolution	Digital Stab
C1	Galaxy S3	3264×2448	1920×1080	off
C2	Galaxy S3 Mini	2560×1920	1280×720	off
C3	Galaxy S3 Mini	2560×1920	1280×720	off
C4	Galaxy S4 Mini	3264×1836	1920×1080	off
C5	Galaxy Tab 3 10.1	2048×1536	1280×720	off
C6	Galaxy Tab A 10.1	2592×1944	1280×720	off
C7	Galaxy Trend Plus	2560×1920	1280×720	off
C8	Ascend G6	3264×2448	1280×720	off
C9	Ipad 2	960×720	1280×720	off
C10	Ipad Mini	2592×1936	1920×1080	on
C11	Iphone 4s	3264×2448	1920×1080	on
C12	Iphone 5	3264×2448	1920×1080	on
C13	Iphone 5c	3264×2448	1920×1080	on
C14	Iphone 5c	3264×2448	1920×1080	on
C15	Iphone 6	3264×2448	1920×1080	on
C16	Iphone 6	3264×2448	1920×1080	on
C17	Lumia 640	3264×1840	1920×1080	off
C18	Xperia Z1c	5248×3936	1920×1080	on

**Table 2:** Rescaling and cropping parameters linking image and video Sensor Pattern Noises (SPNs) for the considered devices, in absence of in-camera digital stabilization.

ID	Scaling	Central Crop along *x* and *y* axes
C1	0.59	[0 307]
C2	0.5	[0 228]
C3	0.5	[0 228]
C4	0.59	[0 0]
C5	1	[408 354]
C6	0.49	[0 246]
C7	0.5	[0 240]
C8	0.39	[0 306]
C9	1	[−160 0]
C17	0.59	[0 1]

**Table 3:** Rescaling and cropping parameters that link image and video SPNs for the considered devices using in-camera digital stabilization. The values are computed on the first 10 frames of the available video reference; min, median (bold), and max values are represented.

ID	Scaling	Central Crop along *x* and *y*	Rotation (CCW)
C10	[0.806 **0.815** 0.821]	[243 **256** 261] [86 **100** 103]	[−0.2 **0** 0.2]
C11	[0.748 **0.750** 0.753]	[380 **388** 392] [250 **258** 265]	[−0.2 **0** 0.2]
C12	[0.684 **0.689** 0.691]	[287 **294** 304] [135 **147** 165]	[−0.2 **0** 0.6]
C13	[0.681 **0.686** 0.691]	[301 **318** 327] [160 **181** 195]	[−0.4 **0** 1]
C14	[0.686 **0.686** 0.689]	[261 **301** 304] [119 **161** 165]	[−0.4 **0** 0]
C15	[0.696 **0.703** 0.713]	[298 **322** 345] [172 **190** 218]	[−0.2 **0.2** 1.6]
C16	[0.703 **0.706** 0.708]	[315 **323** 333] [178 **187** 201]	[−0.2 **0.2** 0.4]
C18	[0.381 **0.384** 0.387]	[548 **562** 574] [116 **121** 126]	[0 **0** 0]

**Table 4:** Performance of Source Identification of digitally stabilized video (using ffmpeg) using both non-stabilized and stabilized references reported in [10], in terms of True Positive Rate (TPR) and False Positive Rate (FPR).

Reference	Query	TPR [10]	FPR [10]
Non-stabilized	Stabilized	0.83	0
Stabilized	Stabilized	0.65	0

**Table 5:** Performance of the proposed method for different values of the aggregation threshold τ.

Aggregation Threshold (τ)	Accuracy	TPR	FPR
30	89%	0.79	0.02
32	89%	0.82	0.05
34	90%	0.84	0.03
36	93%	0.87	0.01
38	93%	0.86	0
40	93%	0.87	0.01
42	93%	0.85	0
44	93%	0.85	0
46	93%	0.85	0
48	92%	0.84	0
50	92%	0.83	0
52	91%	0.82	0
54	91%	0.82	0

**Table 6:** Performance of the proposed method for different values of the aggregation threshold τ.

Test Case	Max PCE	Scale
Zoom only (1080p video)	$2.696\times 10^{4}$	1.3085
Zoom and YouTube @1080p	$5.64\times 10^{2}$	1.3085
Zoom and YouTube @720p	$6.14\times 10^{2}$	0.8722
